# Norwogonin attenuates hypoxia-induced oxidative stress and apoptosis in PC12 cells

**DOI:** 10.1186/s12906-020-03189-8

**Published:** 2021-01-07

**Authors:** Linlin Jing, Rongmin Gao, Jie Zhang, Dongmei Zhang, Jin Shao, Zhengping Jia, Huiping Ma

**Affiliations:** Department of Pharmacy, the 940th Hospital of Joint Logistics Support force of PLA, Lanzhou, 730050 Gansu China

**Keywords:** Norwogonin, Antioxidant activity, Hypoxia, Oxidative stress, Apoptosis

## Abstract

**Background:**

Norwogonin is a natural flavone with three phenolic hydroxyl groups in skeletal structure and has excellent antioxidant activity. However, the neuroprotective effect of norwogonin remains unclear. Here, we investigated the protective capacity of norwogonin against oxidative damage elicited by hypoxia in PC12 cells.

**Methods:**

The cell viability and apoptosis were examined by MTT assay and Annexin V-FITC/PI staining, respectively. Reactive oxygen species (ROS) content was measured using DCFH-DA assay. Lactate dehydrogenase (LDH), malondialdehyde (MDA) and antioxidant enzyme levels were determined using commercial kits. The expression of related genes and proteins was measured by real-time quantitative PCR and Western blotting, respectively.

**Results:**

We found that norwogonin alleviated hypoxia-induced injury in PC12 cells by increasing the cell viability, reducing LDH release, and ameliorating the changes of cell morphology. Norwogonin also acted as an antioxidant by scavenging ROS, reducing MDA production, maintaining the activities of superoxide dismutase (SOD), catalase (CAT) and glutathione peroxidase (GPx), and decreasing the expression levels of HIF-1α and VEGF. In addition, norwogonin prevented cell apoptosis via inhibiting the expression levels of caspase-3, cytochrome c and Bax, while increasing the expression levels of Bcl-2 and the ratio of Bcl-2/Bax.

**Conclusions:**

Norwogonin attenuates hypoxia-induced injury in PC12 cells by quenching ROS, maintaining the activities of antioxidant enzymes, and inhibiting mitochondrial apoptosis pathway.

## Background

Aerobic organisms need oxygen (O_2_) for producing energy. Hypoxia is defined as insufficient O_2_ supply to maintain cellular function in tissue and often occurs in some physiological situations such as high altitude [1], and in many pathological situations such as stroke [2]. The brain is particularly sensitive to hypoxia-induced injury due to its high oxygen consumption, rich in unsaturated fatty acids and low antioxidant capacity [3]. Increasing evidences have indicated that hypoxia can induce adverse effects on brain [4–6].

Oxidative stress and apoptosis are considered as two contributing factors in hypoxia-induced injury [7, 8]. Hypoxia exposure has been reported to increase the production of intracellular reactive oxygen species (ROS), which facilitates oxidative stress. Excessive ROS, such as superoxide anion (O_2_^−˙^), hydrogen peroxide (H_2_O_2_) and hydroxyl radical (HO•), leads to structural and functional cellular changes by attacking lipids, membranes, proteins and DNA, and subsequently causes cell damage [9]. Simultaneously, overproduced ROS also facilitates opening of mitochondrial permeability transition pore (mPTP) [10] and transferring pro-apoptosis proteins to the outer mitochondrial membrane, which induces depolarization of mitochondrial membranes and releases of cytochrome c [11]. These changes ultimately cause mitochondrial-dependent apoptosis [12]. So, it is believed that antioxidant with the ability of inhibiting or eliminating excessive ROS may exert its protective effect via attenuating oxidative stress and apoptosis induced by hypoxia. Lots of studies have proved that antioxidant supplement like vitamin C [13], isoflavone [8] and nitroxide radicals [14], can limit hypoxia-induced injury in vitro and in vivo.

Flavonoids are large and diverse class of ubiquitous plant secondary metabolites. They are always considered as excellent natural antioxidant with the ability of scavenging free radical and inhibiting lipid peroxidation. Currently, more and more attentions have been paid on this class of compounds due to their benefit effects on human health. Flavonoids have been shown to own a wide range of pharmacological actions, such as antiinflammatory, antinociceptive and neuroprotective activity, etc., all which may be attribute to their antioxidant activities [15]. Many studies have indicated that flavonoids exhibit excellent protective effects on hypoxia-induced failure. For example, rutin has a strong neuroprotective effect against retinal ganglion cell death induced by hypoxia [16]. A recent study also demonstrates that rutin can alleviate cobalt chloride-induced hypoxia damage by inhibiting oxidative stress and apoptosis in H9c2 cell [17]. Moreover, Liu et al suggest that nobiletin (3′,4′,5,6,7,8-hexamethoxyflavone) attenuates myocardial I/R injury via activating of Akt/GSK-3β pathway in H9c2 cell [18]. In addition, acacetin can protect rat cardiomyocytes and H9C2 cardiomyoblasts against hypoxia/reoxygenation induced injury via AMPK-mediated activation of Nrf2 signaling pathway [19].

**Fig. 1:**
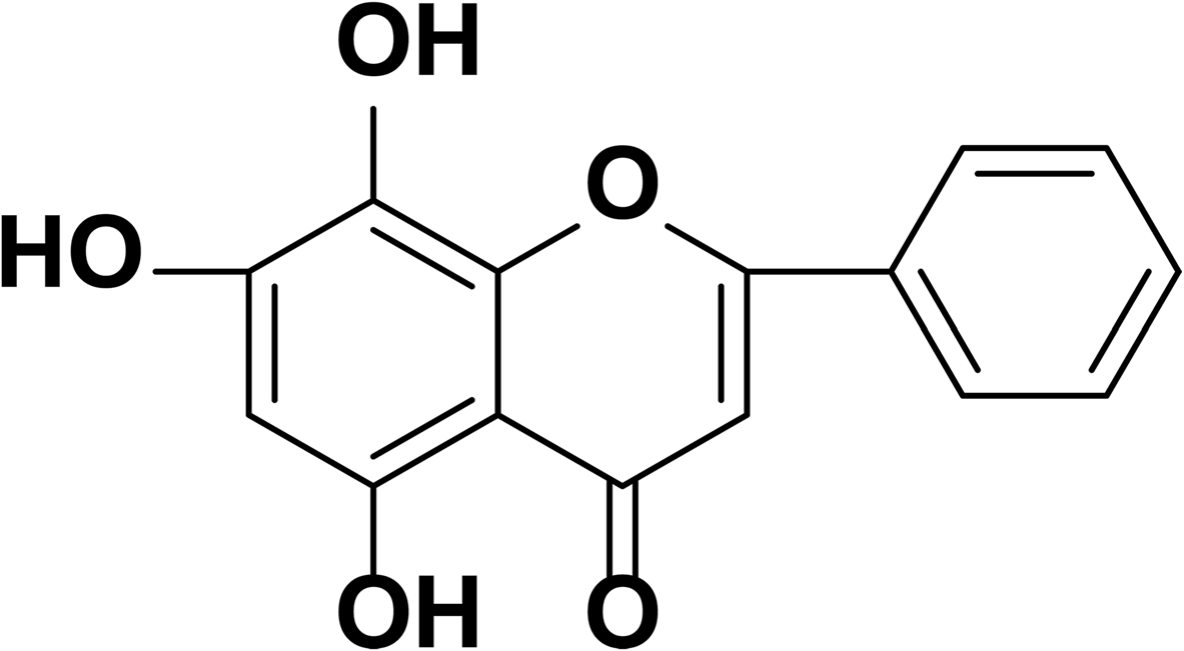
Chemical structure of norwogonin

Norwogonin (5,7,8-trihydroxyflavone, Fig. 1) is a pharmacologically active flavone separated from the root of *Scutellaria baicalensis* Georgi (“Huang Qin” in Chinese), a traditional Chinese herb used to treat fluenza and cancer [20, 21]. However, limited studies have been reported on the biological activities of norwogonin due to its low levels in natural plants. In order to address this problem, several synthesis methods of norwogonin are reported [22, 23]. Our previous study also established a simple method for obtaining norwogonin from chrysin in four steps [24]. These researches have positively influenced the further evaluation of norwogonin′s biological activities.

Studies have revealed that norwogonin owns antioxidant [25], anticancer [26, 27], antiviral [28] and antimicrobial activities [29] as well as inhibits the cyanide-stimulated production of ROS [30]. However, whether norwogonin has protective capacities against hypoxia-induced injury remains unknown. The aim of present study was to investigate the protective effects of norwogonin against hypoxia-induced oxidative stress and apoptosis in PC12 cells.

## Methods

### Materials and reagents

Norwogonin (purity≥98%) was synthesized according to our previous reported method [24]. Rutin (purity≥96%) was purchased from Ci Yuan Biotechology Co., Ltd. (Xian, Shannxi, China). Norwogonin and rutin was dissolved in sterile dimethyl sulfoxide (DMSO), stored at − 20 °C, and diluted in the cell culture medium immediately before using.

Dulbecco’s modified Eagle’s medium (DMEM), fetal bovine serum (FBS), streptomycin and penicillin were purchased from Solarbio co., Ltd. (Beijing, China).

The kits of malondialdehyde (MDA), lactate dehydrogenase (LDH), superoxide dismutase (SOD), catalase (CAT) and glutathione peroxidase (GPx) were obtained from Nanjing Jiancheng Bioengineering Institute (Jiangsu, China). 2′,7′-dichlorodi-hydrofluorescein diacetate (DCFH-DA) and (3-(4,5-dimethylthiazol-2-yl)-2,5-diphenyltetrazolium bromide) tetrazolium (MTT) was obtained from Sigma-Aldrich Co (St. Louis, MO, USA). Primary antibodies for hypoxia inducible factor-1α (HIF-1α), vascular endothelial growth factor (VEGF), B cell lymphoma-2 (Bcl-2), Bcl-2 associated X protein (Bax), Caspase-3, Cytochrome C and *β*-actin were all purchased from Abcam (Cambridge, UK). Secondary antibodies were obtained from ZsBio Company (Beijing, China). Apoptosis analysis kit was obtained from Beyotime Institute of Biotechnology (Jiangsu, China). All chemicals and solvents were of analytical grade and were obtained from commercial supplier in China.

### Cell culture

The PC12 cells were purchased from Cell Bank of the Chinese Academy of Sciences (TCR 9, Shanghai, China) and maintained in DMEM with 10% (v/v) FBS, 100 U/mL penicillin, and 100 U/mL streptomycin at 37 °C in a humidified incubator containing 5% CO_2_.

To evaluate the cytotoxicity of norwogonin, PC12 cells (passage 4 ~ 6) were pre-incubated with different concentrations (10^− 8^, 10^− 7^, 10^− 6^, 10^− 5^, 10^− 4^ mol/L) of norwogonin for 1 h and then cultured for 24 h.

### Hypoxia exposure

To induce cell hypoxia injury model, PC12 cells were subjected to hypoxia environment (1% O_2_, 5% CO_2_, and 94% N_2_) at 37 °C for 24 h in a humidified chamber. Normoxic control cells were cultured at 37 °C in a 5% CO_2_ incubator for 24 h.

To evaluate the protective effect of norwogonin against hypoxia-induced injury, PC12 cells were pre-incubated with different concentrations (10^− 8^, 10^− 7^, 10^− 6^, 10^− 5^ mol/L) of norwogonin for 1 h before hypoxia treatment.

### Cell viability

The cells viability was measured by MTT assay as previously described [31]. In brief, PC12 cells (1 × 10^5^ cells/mL) were seeded in 96 well culture plates. Then different concentrations of norwogonin were added to the wells. Equal volume of DMSO was added to control wells. The final concentration of DMSO in the cell culture medium is 0.1%. After incubation at normoxic or hypoxia condition, 10 μL of MTT (5.0 mg/mL) was added to each well, followed by incubation at 37 °C for 4 h. Then, the supernatant with MTT was removed and the formazan product was dissolved in 100 *μ*L DMSO. The absorbance was measured on a SpectraMax i3 microplate reader (Molecular Devices, Sunnyvale, CA, USA) at 570 nm. The results were expressed as the relative percentage of control group.

### Hematoxylin and eosin (HE) staining

PC12 cells seeded on glass coverslips were incubated for 24 h before they were treated with norwogonin in the same way as described above. The medium was removed and the glass coverslips were washed with cold PBS, followed by fixation with methanol for 10 min at room temperature and then washed with cold PBS three times for 5 min. Finally the cells were stained according to the HE staining protocol [32]. The analyses of the cell were performed using an OLYMPUS IX73 microscope (100×) in order to verify cell morphological changes. Digital images were obtained using the DXM 1200 C digital camera (Nikon) associated to the microscope.

### ROS content

Intracellular ROS level in PC12 cells was determined using DCFH-DA assay [33]. Briefly, PC12 cells (1 × 10^5^ cells/mL) were seeded in 6-well plates. After hypoxia treatment, PC12 cells were washed with PBS, and then were incubated in the culture medium containing 10 μM DCFH-DA for 30 min in the dark at 37 °C. The cells were observed with Olympus inverted fluorescence microscope (Tokyo, Japan) and were analyzed by a Becton Dickinson FACScan flow cytometer (BD Biosciences, CA, USA) with excitation wavelength of 488 nm and emission wavelength of 525 nm. The ROS level was expressed as relative percentage of control.

### LDH leakage, MDA content and antioxidant enzyme activity

PC12 cells (1 × 10^5^ cells/mL) were seeded in 90 mm dish. After hypoxia treatment as described above, 50 μL culture supernatant from each dish were collected, and LDH activity in medium was detected using commercial assay kits (Jiancheng Institute of Biotechnology, Nanjing, China) and was expressed as U/mL. The PC12 cells were harvested and homogenized after washing two times with cold PBS. The concentration of total protein was measured by BCA protein assay kit. The MDA content and antioxidant enzyme activity were determined using commercial assay kits (Jiancheng Institute of Biotechnology, Nanjing, China). The content of MDA was presented as nmol/mg protein. The activities of SOD, CAT and GPx were presented as U/mg protein.

### Cell apoptosis(Annexin-V/PI staining)

After hypoxia treatment, PC12 cells were harvested, washed two times with cold PBS, and then suspended with binding buffer. The cells were treated with the Annexin V-FITC and PI solution following the protocol of the manufacturer (Beyotime, Shanghai, China). Data collections were performed using Becton Dickinson FACScan flow cytometer (BD Biosciences, CA, USA).

### Quantitative real-time PCR analysis

The total RNA of PC12 cells was extracted using Trizol reagent (Takara, Dalian, China) and converted to cDNA using the PrimeScript TM RT reagent Kit (AK4301, Takara, Dalian, China). The cDNA encoding HIF-1α, VEGF, Bcl-2, Bax, caspase-3, cytochrome C and glyceraldehyde-3-phosphate dehydrogenase (GAPDH) gene was amplified by Quantitative real-time PCR using a 7300 real-time detection System (Applied Biosystems, CA, USA). The primers used were shown in Table 1. The PCR cycling conditions were 95 °C for 30 s, follow by 40 cycles of 95 °C for 5 s and 60 °C for 31 s. The mRNA levels were calculated using the 2^-ΔΔCt^ method and normalized to GAPDH, which as the reference gene.

**Table 1:** Primers used in real-time qPCR

Gene	Primer sequences	Product (bp)
HIF-1*α*	forward: 5′-CCAGATTCAAGATCAGCCAGCA-3′ reverse: 5′-GCTGTCCACATCAAAGCGTATA-3´	100
VEGF	forward:5´-ACATTGGCTCACTTCCAGAAACA-3′ reverse:5-TGGTTGGAACCGGATCTTTA-3´	108
Bcl-2	forward:5′-GGTGGTGGAGAACTCTTCACGT-3′ reverse:5´-AGGATTGTGGCTGAACA-3´	253
Bax	forward: 5´-TGGCGATGAACTGGACAACAA-3′ reverse: 5′-GGGAGTCTGTATCCACATCAGCA-3´	65
Caspase-3	forward:5´-AGACAGACAGTGGAACTGACGATG-3′ reverse:5′-GGCGCAAAGTGACTGGATGA-3´	147
Cytochrome C	forward:5-GAAGAAGGGAGAAAGGGCAGA-3′ reverse:5′-CGGGGGCTGTCCAACAAA-3´	302
GAPDH	forward: 5´-GCCACAGTCAAGGCTGAGAATG-3′ reverse:5′-ATGGTGGTGAAGACGCCAGTA-3´	143

### Western blot

PC12 cells were harvested and homogenized in RIPA agents. The concentration of total proteins was quantified using BCA protein assay kit. 30 μg of samples were resolved on 12% SDS-PAGE electrophoresis and then transferred to polyvinylidene fluoride (PVDF) membranes (Millipore, Billerica, MA, USA). The membranes were blocked with 5% non-fat dry milk in TBST buffer for 1 h at room temperature and incubated with primary antibodies: anti-HIF-1*α* (1:300, ab179483, Abcam, UK), anti-VEGF (1:1000, ab46154, Abcam, UK), anti-Bcl-2 (1:1000, ab59348, Abcam, UK), anti-Bax (1:500, ab32503, Abcam, UK), anti-caspase-3 (1:300, ab44976, Abcam, UK), anti-cytochrome C (1:1000, ab13575, Abcam, UK) and anti-*β*-actin (1:2000, ab8227, Abcam, UK) at 4 °C overnight. Then, the membranes were washed and incubated with secondary antibodies (1:2000, ZsBio, Beijing, China;) for 1 h at room temperature. The immunoreactive bands were visualized using enhanced chemiluminescence (ECL) reagents. The relative intensities of bands were normalized to the *β*-actin internal control and analyzed using Image-Pro Plus 6.0 (Media Cybernetics, Inc., Bethesda, MD, USA).

### Statistical analysis

The results were expressed as mean ± SD derived from at least three independent experiments. Difference between groups were analyzed using one-way analysis of variance (ANOVA) following by Student–Newman–Keuls post hoc test. A *P*-value of < 0.05 was regarded as statistically significant.

## Results

### Norwogonin protective PC12 cells against hypoxia-induced injury

**Fig. 2:**
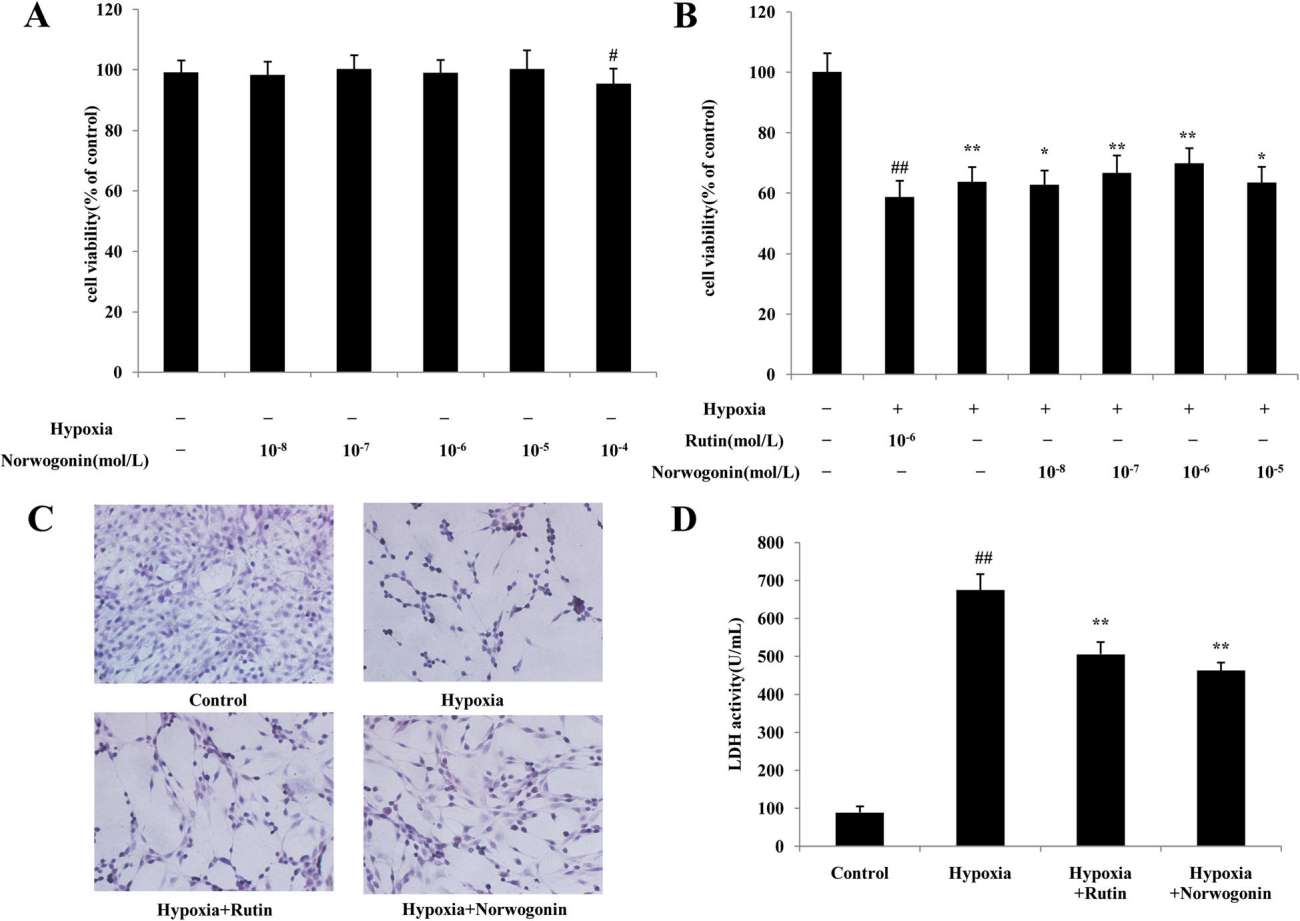
Norwogonin inhibited hypoxia-induced injury in PC12 cells. MTT assay (**a** and **b**). PC12 cells were treated with variable concentrations of norwogonin for 1 h, and then incubated for another 24 h under normal (**a**) or hypoxia (**b**) condition. Representative microscopy (**c**) and LDH leakage (**d**). Cells were incubated with norwogonin for 1 h and then exposed to hypoxia for 24 h. After the treatment, the cell was stained with hematoxylin and eosin and examined under light microscopy. The levels of LDH release in cell suspensions were analyzed by the LDH assay kit. Results are expressed as means ± SD, *n* = 6 or 3. ^##^*P* < 0.01 vs control group, ^*^P < 0.05, ^**^P < 0.01 vs hypoxia group

First, to preclude the proliferative activity of norwogonin, its cytotoxicity on normal PC12 cells was determined using MTT assay. As seen in Fig. 2a, cellular proliferation was not significantly changed following treatment with norwogonin at concentrations of 1 × 10^− 8^-1 × 10^− 5^ mol/L (*P* > 0.05). However, cell viability significantly decreased when the concentration of norwogonin was increased to 1 × 10^− 4^ mol/L (*P* < 0.05). The results indicated that norwogonin did not exhibit toxicity or proliferative activity on PC12 cells at the concentrations of 1 × 10^− 8^-1 × 10^− 5^ mol/L.

Then we examined the protective effect of norwogonin against hypoxia-induced PC12 cells injury. As shown in Fig. 2b, compared with the control group, the cell viability in hypoxia group was decreased to 58.71% (*P* < 0.01). Compared with the hypoxia treatment, pretreated with 1 × 10^− 8^, 1 × 10^− 7^, and 1 × 10^− 6^ mol/L norwogonin dose-dependently protected PC12 cells against hypoxia-induced injury, recovering the cell viability from 58.71 to 62.79% (*P* < 0.05), 66.68% (*P* < 0.01) and 69.88% (*P* < 0.01), respectively. Pretreatment with 1 × 10^− 6^ mol/L rutin also exhibited protective effect, significantly increasing the cell viability to 63.78% compared to hypoxia treatment. The viability pretreated with 1 × 10^− 5^ mol/L norwogonin was decreased to 63.43%, which still significant higher than that in hypoxia group (*P* < 0.05). These results demonstrated that norwogonin showed significant cytoprotection at the concentrations of 1 × 10^− 8^-1 × 10^− 5^ mol/L and the most effective concentration is 1 × 10^− 6^ mol/L. Then this dose was used as optimal dose in the following experiments.

The protective capacity of norwogonin was also verified by the morphological alterations. As shown in Fig. 2c, PC12 cells without hypoxia treatment grew well with regular shapes (fusiform), uniform sizes. After hypoxia exposure, PC12 cells exhibited shrinkage, rounded shape, desquamation, and reduced cell density. The cells pretreated with norwogonin or rutin before hypoxia exposure grew better, the number of desquamation cells decreased, and cell shape recovered normally.

Besides, the protective ability of norwogonin was confirmed by the LDH leakage, which is associated with the loss of cell-membrane integrity. As shown in Fig. 2d, the LDH activity in culture medium was notably increased following hypoxia exposure (*P* < 0.01). Pretreatment with norwogonin or rutin dramatically decreased the LDH leakage, suggesting norwogonin and rutin restored the cell-membrane integrity.

### Norwogonin inhibits hypoxia-induced oxidant stress in PC12 cells

**Fig. 3:**
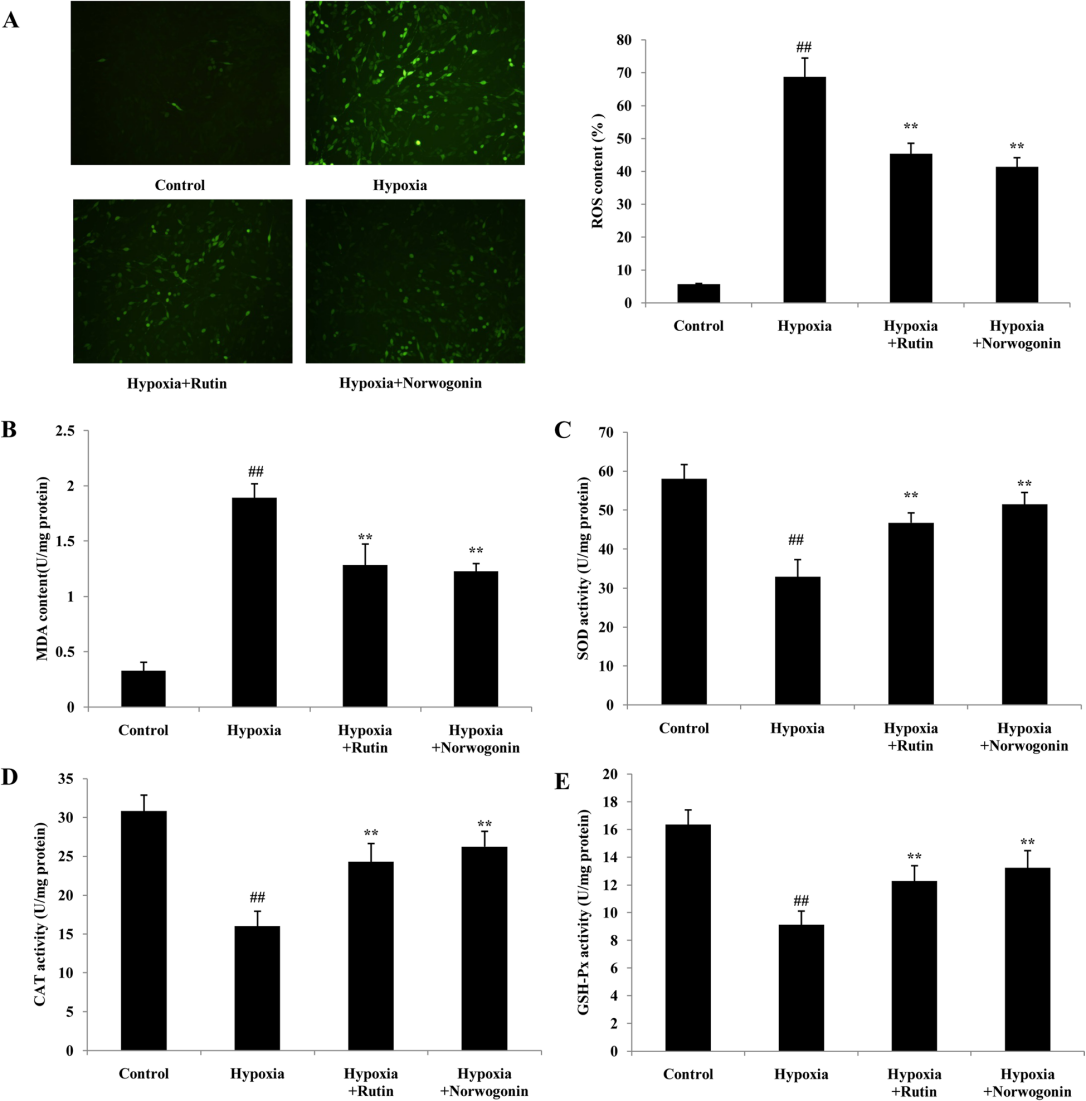
Norwogonin suppressed hypoxia-induced oxidant stress in PC12 cells. Cells were incubated with norwogonin for 1 h and then exposed to hypoxia for 24 h. After the treatment, ROS level (**a**) in cell was measured using DCF fluorescence assay, MDA content (**b**), SOD (**c**), CAT (**d**) and GPx (**e**) activities were analyzed by commercial assay kit. Results are expressed as means ± SD, *n* = 3. ^##^*P* < 0.01 vs control group, ^*^P < 0.05, ^**^P < 0.01 vs hypoxia group

ROS and MDA are two important indicators of cellular oxidant stress induced by hypoxia. As shown in Fig. 3a and b, a significant increased content of ROS and MDA was observed in PC12 cells following hypoxia exposure. Norwogonin or rutin pretreatment significantly inhibited the production of ROS and MDA. Antioxidant enzymes, such as SOD, CAT, and GPx, are regarded as the main defense system against oxidative stress in cell. As shown in Fig. 3c-e, hypoxia exposure significantly inhibited the activities of SOD, CAT, and GPx in PC12 cells. Treatment with norwogonin or rutin reversed these changes and restored the activities of antioxidant enzymes. All these results indicated that norwogonin protected the PC12 cells against oxidative stress induced by hypoxia.

### Norwogonin inhibits the increased HIF-1α and VEGF expressions in PC12 cells under hypoxia

**Fig. 4:**
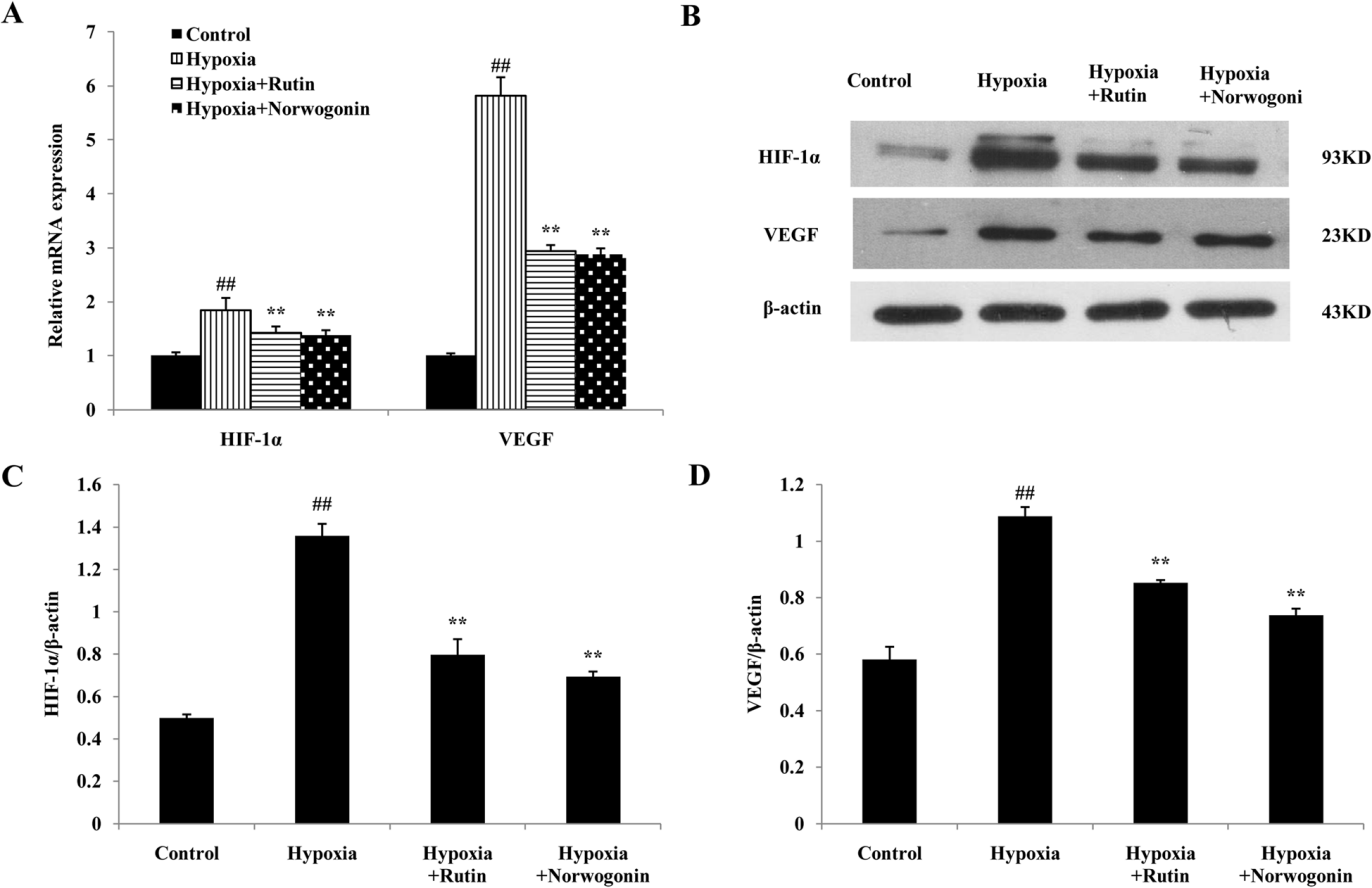
Norwogonin down-regulated the expression of HIF-1*α* and VEGF in PC12 cells following hypoxia exposure. Cells were incubated with norwogonin for 1 h and then exposed to hypoxia for 24 h. The mRNA expression was determined by the real-time qPCR (**a**). The expressions of HIF-1α and VEGF proteins were detected using Western blot analysis (**b**). In order to improve the clarity and conciseness of the presentation, the blots were cropped. Statistical analysis results of HIF-1α (**c**) and VEGF (**d**) from Western blot analysis. Results are expressed as means ± SD, n = 3. ^##^*P* < 0.01 vs control group, ^*^P < 0.05, ^**^P < 0.01 vs hypoxia group

As seen in Fig. 4a, the expressions of HIF-1*α* and VEGF mRNA in PC12 cells were increased significantly following hypoxia exposure. These changes were inhibited by norwogonin or rutin pretreatment. Similarly, compared to the control group, the expressions of HIF-1*α* and VEGF protein were significantly increased in hypoxia group. However, pretreatment of norwogonin or rutin significantly downregulated the protein expression levels of HIF-1*α* and VEGF (Fig. 4b-d).

### Norwogonin inbibits hypoxia-induced apoptosis in PC12 cells

**Fig. 5:**
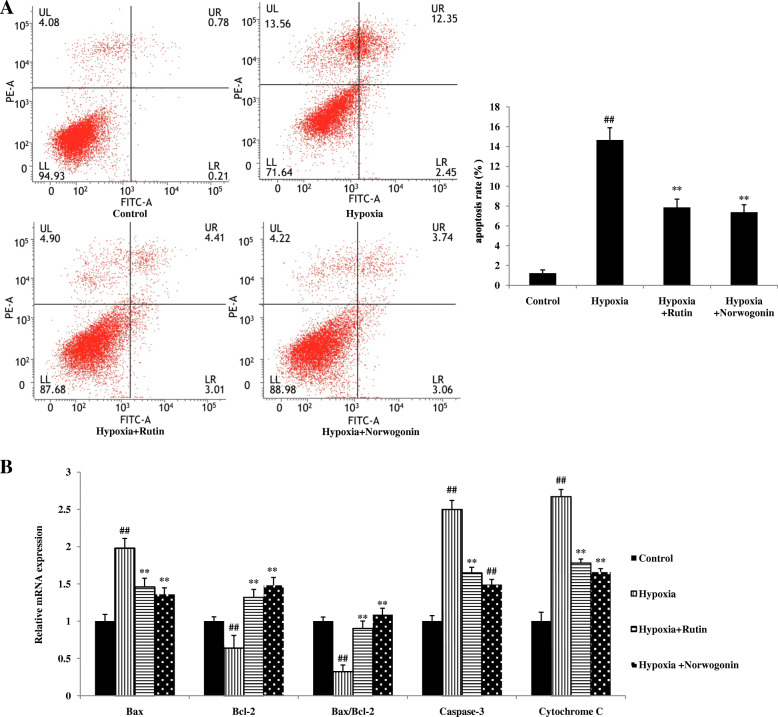
Norwogonin alleviated hypoxia-induced cell apoptosis and regulated the expression of apoptosis-related gene in PC12 cells. Cells were incubated with norwogonin for 1 h and then exposed to hypoxia for 24 h. After the treatment, the staining cells with AnnexinV-FITC and PI labeling were analyzed by flow cytometric (**a**). The mRNA expression was determined by the real-time qPCR (**b**). Results are expressed as means ± SD, n = 3. ^##^*P* < 0.01 vs control group, ^*^P < 0.05, ^**^P < 0.01 vs hypoxia group

The effects of norwogonin on hypoxia-induced apoptosis were performed by FCM. As seen in Fig. 5a, compared to the normal group, the apoptosis rate of PC12 cells was significantly increased after hypoxia exposure for 24 h. However, norwogonin and rutin significantly inhibited hypoxia-induced apoptosis with the evidence by decreased apoptosis rate of PC12 cells in norwogonin and rutin groups compared with hypoxia group.

**Fig. 6:**
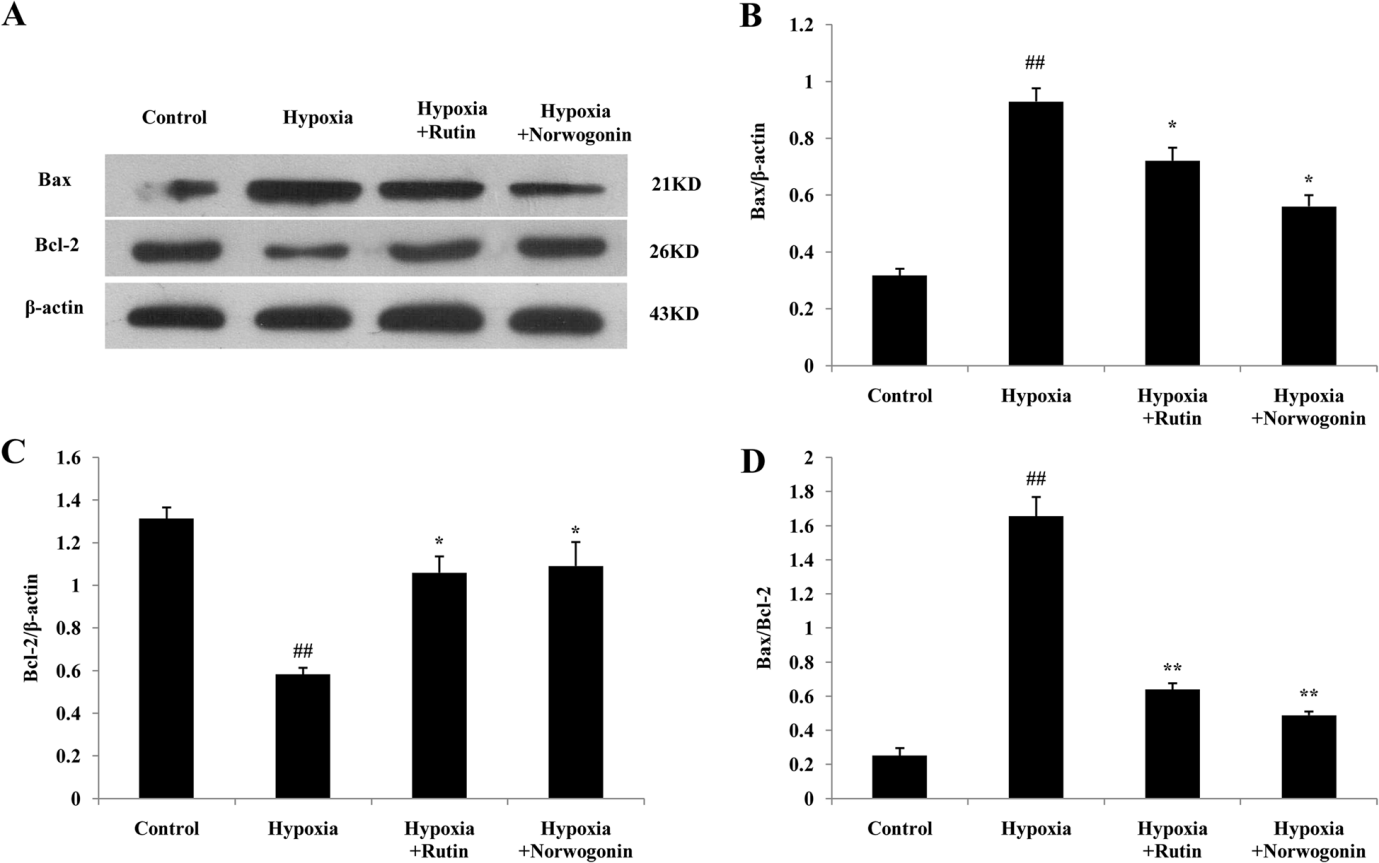
Norwogonin regulated the expression of apoptosis-related proteins in PC12 cells. Cells were incubated with norwogonin for 1 h and then exposed to hypoxia for 24 h. The expressions of Bax and Bcl-2 proteins were detected using Western blot analysis (**a**). In order to improve the clarity and conciseness of the presentation, the blots were cropped. Statistical analysis results of Bax (**b**), Bcl-2 (**c**) and Bax/Bcl-2 (**d**) from Western blot analysis. Results are expressed as means ± SD, n = 3. ^##^*P* < 0.01 vs control group, ^*^P < 0.05, ^**^P < 0.01 vs hypoxia group

**Fig. 7:**
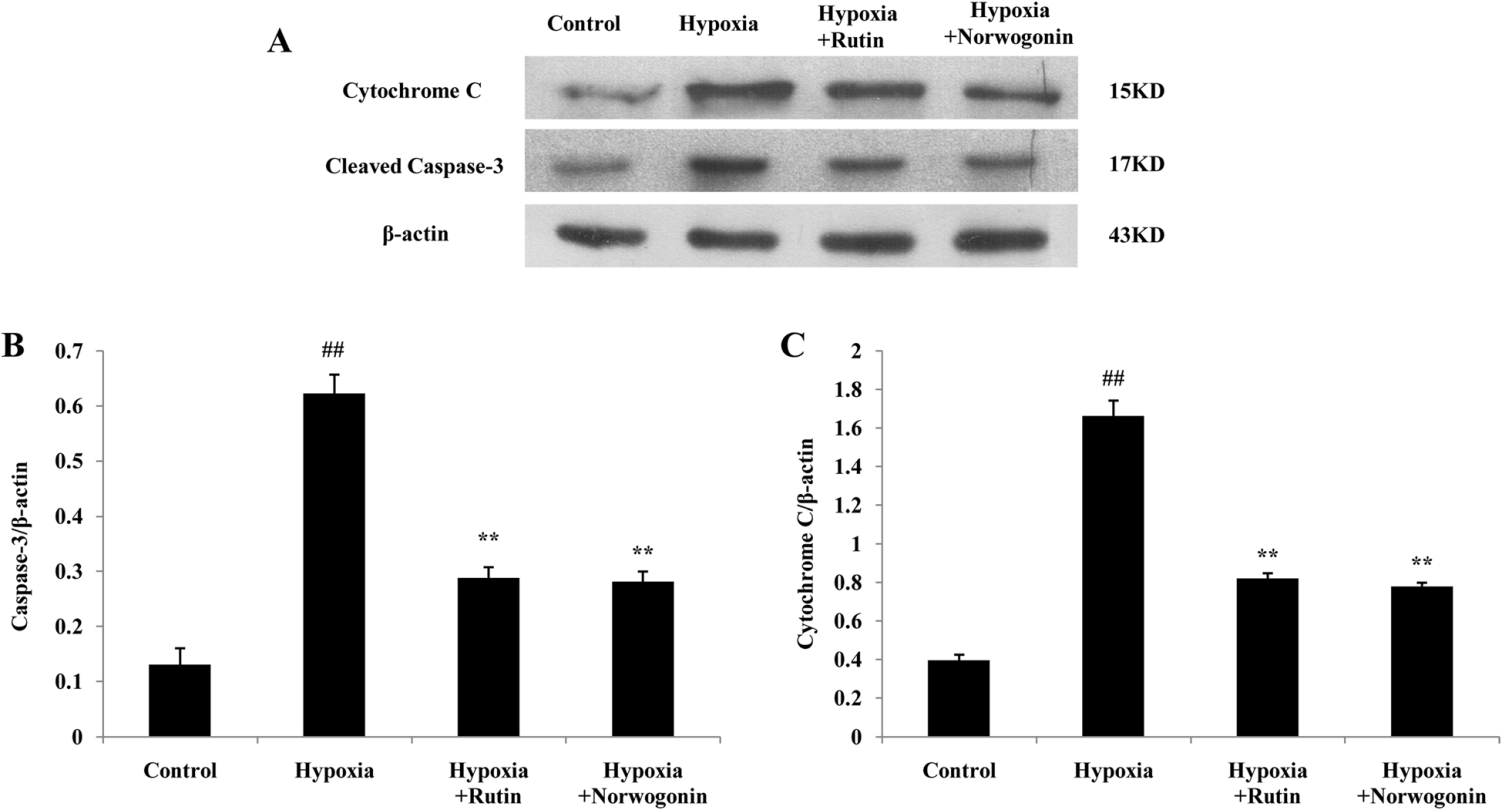
Norwogonin decreased the expression of Cytochrome c and Caspase-3 in PC12 cell following hypoxia exposure. Cells were incubated with norwogonin for 1 h and then exposed to hypoxia for 24 h. The expressions of Cytochrome c and Caspase-3 proteins were detected using Western blot analysis (**a**). In order to improve the clarity and conciseness of the presentation, the blots were cropped. Statistical analysis results of Caspase-3 (**b**) and Cytochrome c (**c**) from Western blot analysis. Results are expressed as means ± SD, n = 3. ^##^*P* < 0.01 vs control group, ^*^P < 0.05, ^**^P < 0.01 vs hypoxia group

To further confirm the anti-apoptotic effect and mechanisms of norwogonin against hypoxia-induced injury, the expressions of apoptosis-related genes and proteins, such as Bcl-2, Bax, cytochrome c and caspase-3, were detected using RT-PCR and western blot analysis. As shown in Fig. 5b, compared with control group, hypoxia exposure significantly increased the level of Bax mRNA and the ratio of Bax/Bcl-2, and significantly decreased the level of Bcl-2 mRNA in PC12 cells. In contrast, pretreatment with norwogonin or rutin could reverse these effects. Additionally, hypoxia exposure greatly increased the level of cytochrome c mRNA, which was related to mitochondrial dysfunction, while norwogonin and rutin abolished the increased expression level of cytochrome c mRNA induced by hypoxia. Furthermore, hypoxia exposure up-regulated the level of caspase-3 mRNA in PC12 cells, which was also blocked by norwogonin or rutin pretreatment. Consistently, changes of Bcl-2, Bax, cytochrome c and caspase-3 protein expression levels were similar to the trend of mRNA changes (Fig. 6 and Fig. 7).

## Discussion

Recently, much attention has been paid on looking for novel neuroprotective agents with excellent activity and low adverse effects from naturally occurring products [34]. Flavones are one of the largest and widely natural products distributed in plant kingdom [35] and have been extensively used in the pharmaceutical, chemical and nutraceutical industry. It is well known that the free radical scavenging activity of flavones contribute mainly to the presence of hydroxyl groups [36]. Norwogonin owns three phenolic hydroxyl groups (two of them are consecutive hydrogen group) in structure and may have excellent antioxidant activity. Previous study found that norwogonin had weak effect on inhibiting lipopolysaccharide (LPS)- or lipoteichoic acid (LTA) -induced nitric oxide synthase (iNOS) protein expression and nitric oxide (NO) production [37], but it exhibited better antioxidative activity than acacetin and icariin in protecting human erythrocytes against free-radical-induced haemolysis [25]. Our recent study also demonstrated that norwogonin displayed excellent scavenging activity on 2, 2′-diphenyl-2-picrylhydrazyl (DPPH), O_2_^−˙^and NO in vitro [38]. But the protective effect of norwogonin on hypoxia-induced neurotoxicity remains unclear. PC12 cells, a rat pheochromocytoma cell line, have been extensively considered as a cell line model for screening neuroprotective drugs. In the current study, we used a hypoxia-induced PC12 cells injury model to evaluate the protective effect of norwogonin.

Increasing evidences have indicated that hypoxia exposure results in cytotoxicity in many different cell types, such as hippocampal cells [39] and cardiomyocyte [40]. Hypoxia-induced injury model of PC12 cells has been established in many studies [41, 42]. In line with previous reports, our present result found that the viability of PC12 cells was significantly decreased following hypoxia exposure for 24 h. However, pretreatment with norwogonin prevented the loss of cell viability. In addition, hypoxia exposure caused obvious morphological changes of the cells, while norwogonin reduced these changes and maintained regular shapes of PC12 cells. Furthermore, norwogonin decreased the LDH leakage, suggesting that norwogonin mitigated the cell membrane damage induced by hypoxia.

It has proved that oxidant stress plays a vital role in hypoxia-induced injury. In present study, ROS and MDA content and antioxidant enzymes activities were used to assess the protective effects of norwogonin on hypoxia-induced oxidative stress in PC12 cells. ROS, generated via the mitochondrial electron transport chain, involves physiological roles in cellular signaling pathways at low concentrations, while excessive ROS levels are known to be harmful to major biomolecules such as DNA, lipids, and proteins in cells. In addition, ROS can interact with polyunsaturated lipids in cell membrane, forming MDA, which is widely accepted as a marker of lipid peroxidation. Normally, endogenous antioxidant enzyme, including SOD, CAT and GPx, can effectively remove ROS. However, during hypoxic exposure, excessive ROS directly damage antioxidant enzymes and reduce their activities, resulting in further aggravating oxidative stress in cell [43]. In line with findings from previous reports, our present study also found that hypoxia exposure caused oxidative stress in PC12 cells as evidenced by enhancing the ROS and MDA level, and by decreasing SOD, CAT and GPx activities. Norwogonin pretreatment significantly inhibited intracellular ROS production, decreased MDA levels, and restored the antioxidant enzymes activity, suggesting that norwogonin could ameliorate oxidative stress induced by hypoxia, which was partly due to its up-regulation of antioxidant enzymes.

HIF-1*α* is one of the essential transcription factors responding to hypoxia in cell [44]. It is well known that HIF-1*α* will be stabilized and increased the expression in hypoxia condition. Some studies have demonstrated that overexpression of HIF-1*α* is protective against hypoxic induced injuries [45, 46]. While other studies indicate that upregulation of HIF-1*α* is also considered as a sign of tissue hypoxia [47]. Abundant evidence has corroborated that ROS produced in the mitochondria are responsible for stabilizing HIF-1α during hypoxia [48]. Therefore, easing the formation ROS, either genetically or pharmacologically, cause downregulation of HIF-*α* in hypoxia. As expected, our present results showed that hypoxia upregulated the expression of HIF-1*α* and VEGF, which was a downstream target gene of HIF-1. However, pretreatment with norwogonin could reverse these changes by scavenging ROS.

It is well known that apoptosis is closely related to hypoxia-induced damage [49]. Consistent with previous reports, the present study also found that hypoxia exposure for 24 h significantly induced PC12 cells apoptosis. Correspondingly, pretreatment with norwogonin reduced cell apoptosis induced by hypoxia. However, our results were inconsistent with another findings that norwogonin effectively induced apoptosis in human leukemia HL-60 cells [26] and in triple-negative breast cancer (TNBC) cells [27]. These contradictory results may be due to the different cell lines and concentration of norwogonin used in the experiments.

Many studies have indicated that hypoxia can induce cell apoptotic via increasing the generation of ROS [50], elevating the expression of HIF1-*α* [51, 52], activating mitochondrial pathway [53, 54], and so on. To further determine whether norwogonin demoted apoptosis via mitochondrial pathway in PC12 cell exposure to hypoxia, the relative levels of apoptosis-related genes and proteins were determined. The bcl-2 family proteins, such as the pro-apoptotic protein Bax and anti-apoptosis protein Bcl-2, play a vital role in the mitochondria-dependent apoptotic pathway [55]. Increasing Bcl-2/Bax ratio leads the release of cytochrome c, which further promotes the activation of caspase-3 and leads to apoptotic cell death [56]. Our data revealed that hypoxia exposure for 24 h activated the mitochondrial apoptosis pathway by upregulating Bax, cytochrome c and cleaved caspase-3 expression and downregulating Bcl-2 expression. However, norwogonin treatment remarkably increased Bcl-2 level and decreased the Bax level in PC12 cells exposed to hypoxia. Furthermore, the increased expressions of cytochrome c and cleaved caspase-3 were also reversed by norwogonin. These results suggested that norwogonin could protect PC12 cells from hypoxia-induced injury via mitochondrial-dependent apoptosis pathway.

## Conclusion

In conclusion, our results firstly suggested that norwogonin exhibited excellent protective effects on hypoxia-induced oxidative stress and apoptosis in PC12 cells by scavenging ROS, maintaining the activity of antioxidant enzymes and inhibiting mitochondrial apoptosis pathway.

## Supplementary Information

**Additional file 1.**

## Data Availability

The datasets used and/or analyzed during the current study available from the corresponding author on reasonable request.

## Abbreviations

ROS: Reactive oxygen species
LDH: Lactate dehydrogenase
MDA: Malondialdehyde
SOD: Superoxide dismutase
CAT: Catalase
GPx: Glutathione peroxidase
HIF-1α: Hypoxia inducible factor-1α
VEGF: Vascular endothelial growth factor
Bcl-2: B cell lymphoma-2
Bax: Bcl-2 associated X protein
Akt: Protein kinase B
AMPK: AMP-activated protein kinase
DMSO: Dimethyl sulfoxide
DMEM: Dulbecco’s modified Eagle’s medium
FBS: Fetal bovine serum
MTT: (3-(4,5-dimethylthiazol-2-yl)-2,5-diphenyltetrazolium bromide) tetrazolium
HE: Hematoxylin-Eosin
DCFH-DA: 2′,7′-dichlorodihydrofluorescein diacetate
BCA: Bicinchoninic acid
RIPA: Radioimmunoprecipitationassay
PVDF: Polyvinylidene fluoride
GAPDH: Glyceraldehyde-3-phosphate dehydrogenase
ECL: Enhanced chemiluminescence
ANOVA: Analysis of variance
DPPH: 2,2′-diphenyl-2-picrylhydrazyl
BNIP3: Bcl-2 interacting protein 3
LPS: Ipopolysaccharide
LTA: Lipoteichoic acid
NO: Nitric oxide
iNOS: Nitric oxide synthase
TNBC: triple-negative breast cancer.
