# Supplementary material for: Norwogonin attenuates hypoxia-induced oxidative stress and apoptosis in PC12 cells
Source: BMC Complement Med Ther. 2021 Jan 7;21:18. doi: 10.1186/s12906-020-03189-8 (PMC7791982; doi:10.1186/s12906-020-03189-8)
Supplement: Supplementary file 1 — Additional file 1. [file 12906_2020_3189_MOESM1_ESM.docx]

**Supplementary Information**

**Figure S1.** Norwogonin down-regulated the expression of HIF-1α and VEGF proteins in PC12 cells following hypoxia exposure. a, Normoxic group; b, Hypoxia group; c, Rutin group; d, Norwogonin group.

**Figure S2.** Norwogonin down-regulated the expression of Bax and Bax/Bcl-2 ratio and up-regulate the expression of Bcl-2 in PC12 cells following hypoxia exposure. a, Normoxic group; b, Hypoxia group; c, Rutin group; d, Norwogonin group.

**Figure S3.** Norwogonin decreased the expression of Cytochrome c and Caspase-3 protein in PC12 cells following hypoxia exposure. a, Normoxic group; b, Hypoxia group; c, Rutin group; d, Norwogonin group.

| HIF-1α | a b c d a b c d |
| --- | --- |
|  | 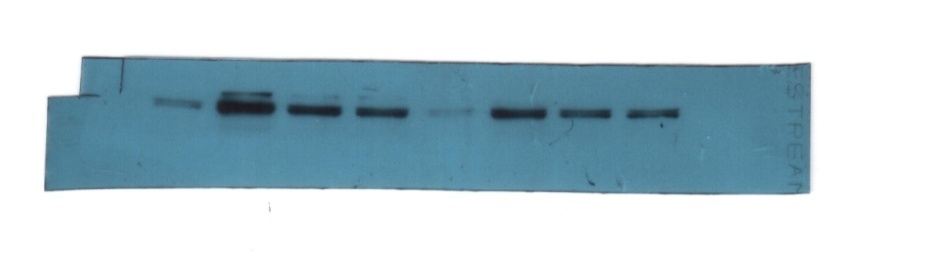 |
|  | a b c d |
|  | 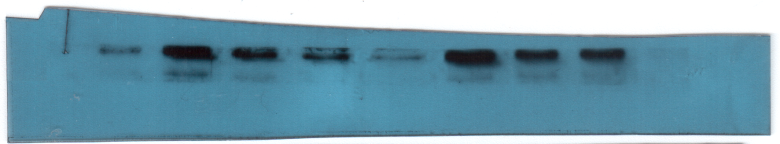 |
| VEGF | a b c d a b c d |
|  | 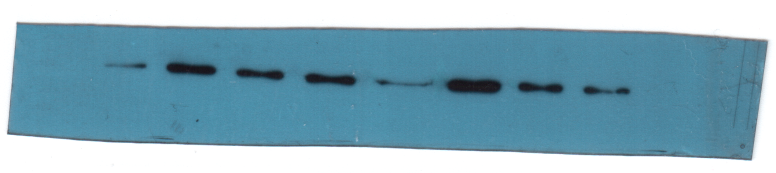 |
|  | a b c d |
|  | 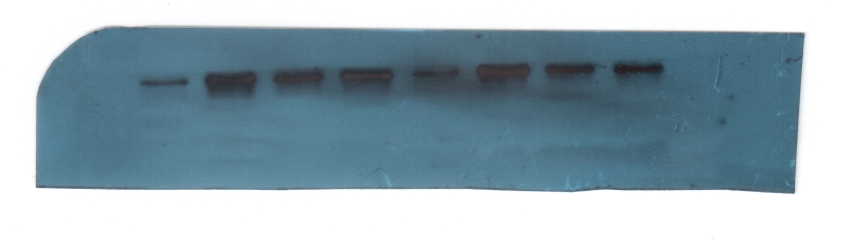 |
| β-actin | a b c d a b c d |
|  | 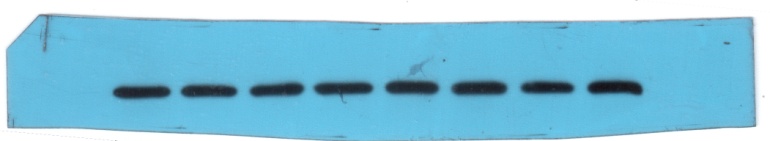 |
|  | a b c d a b c d |
|  | 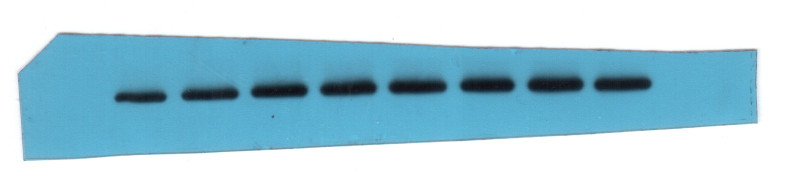 |

**Figure S1.** Norwogonin down-regulated the expression of HIF-1α and VEGF and proteins in PC12 cells following hypoxia exposure. a, Normoxic group; b, Hypoxia group; c, Rutin group; d, Norwogonin group.

| Bax | a b c d a b c d |
| --- | --- |
|  | 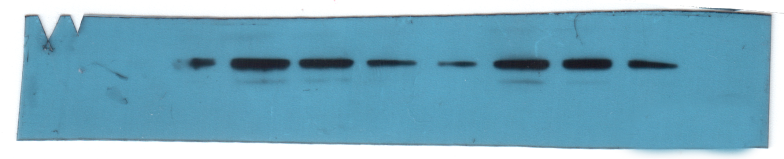 |
|  | a b c d |
|  | 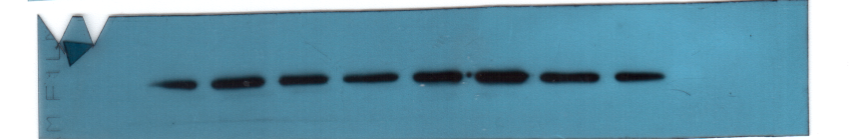 |
| Bcl-2 | a b c d a b c d |
|  | 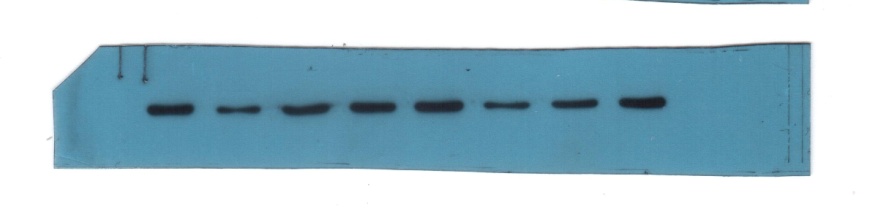 |
|  | a b c d |
|  | 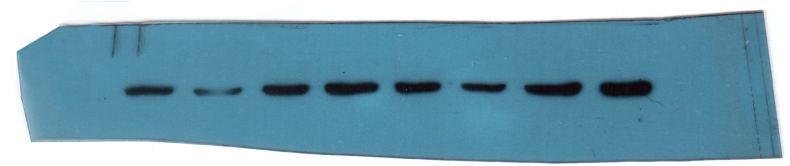 |
| β-actin | a b c d a b c d |
|  | 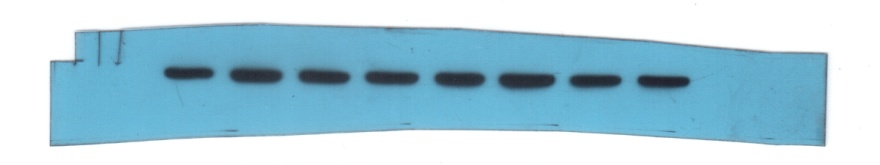 |
|  | a b c d |
|  | 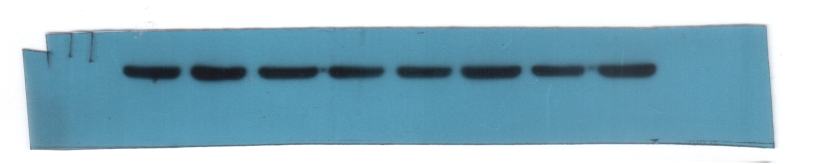 |

**Figure S2.** Norwogonin down-regulated the expression of Bax and Bax/Bcl-2 ratio and up-regulate the expression of Bcl-2 in PC12 cells following hypoxia exposure. a, Normoxic group; b, Hypoxia group; c, Rutin group; d, Norwogonin group.

| Cytochrome c | a b c d a b c d |
| --- | --- |
|  | 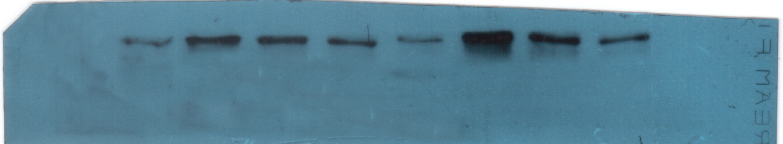 |
|  | a b c d |
|  | 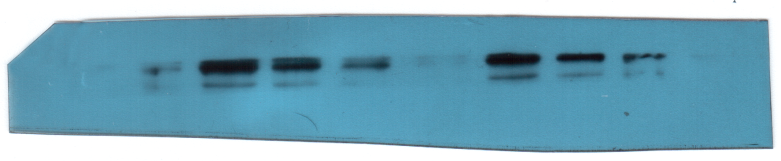 |
| Caspase-3 | a b c d a b c d |
|  | 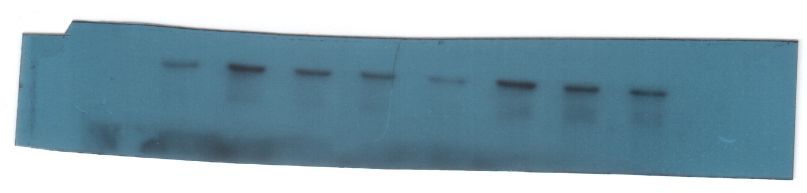 |
|  | a b c d |
|  | 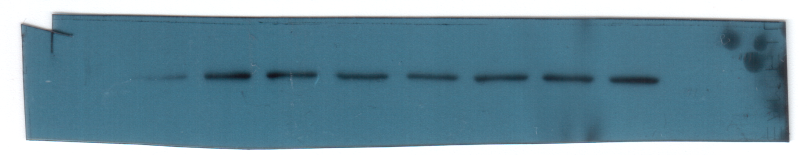 |
| β-actin | a b c d a b c d |
|  | 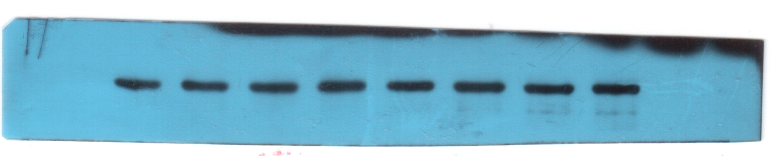 |
|  | a b c d |
|  | 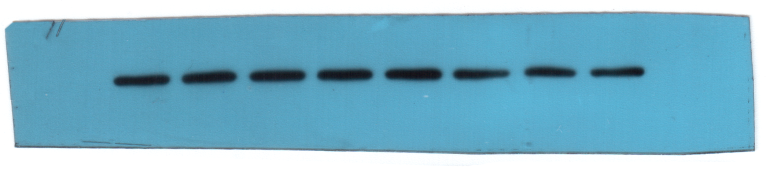 |

**Figure S3.** Norwogonin decreased the expression of Cytochrome c and Caspase-3 protein in PC12 cells following hypoxia exposure. a, Normoxic group; b, Hypoxia group; c, Rutin group; d, Norwogonin group.
